# Advancing Efficiency Sustainability in Poultry Farms through Data Envelopment Analysis in a Brazilian Production System

**DOI:** 10.3390/ani14050726

**Published:** 2024-02-26

**Authors:** Stefanni Marmelstein, Igor Pinheiro de Araújo Costa, Adilson Vilarinho Terra, Ricardo Franceli da Silva, Gabriel Pereira de Oliveira Capela, Miguel Ângelo Lellis Moreira, Claudio de Souza Rocha Junior, Carlos Francisco Simões Gomes, Marcos dos Santos

**Affiliations:** 1 Business Administration Department, Getúlio Vargas Foundation, São Paulo 01331-010, Brazil; stefannimarmelstein@gmail.com; 2 Production Engineering Department, Federal Fluminense University, Rio de Janeiro 24210-240, Brazil; costa_igor@id.uff.br (I.P.d.A.C.); adilsonvterra@gmail.com (A.V.T.); claudiodesouzarochajunior@gmail.com (C.d.S.R.J.); cfsg1@bol.com.br (C.F.S.G.); 3 Economics Department, FEA—University of São Paulo, São Paulo 05508-010, Brazil; ricardo.franceli.silva@faculdadefia.edu.br; 4 Operational Research Department, Brazilian Navy, Rio de Janeiro 20091-000, Brazil; gabrielcapela02@gmail.com; 5 Systems and Computing Department, Military Institute of Engineering, Rio de Janeiro 22290-270, Brazil; marcosdossantos@ime.eb.br

**Keywords:** operational research, data envelopment analysis, integrated production, performance analysis, poultry farming

## Abstract

**Simple Summary:**

This research evaluates the production efficiency of broiler batches using the production efficiency factor and unit cost of production. Employing Data Envelopment Analysis (DEA), it considers variables like poultry housing, age at slaughter, feed consumed, mortality, and unit cost, with the total available weight as the output. Among 31 decision-making units (DMUs), only DMU 4 and DMU 23 approached maximum efficiency. Efficient DMUs serve as benchmarks for disseminating best practices to less efficient ones, enhancing overall efficiency and financial sustainability in poultry farming. This study highlights the significance of unit cost in evaluating production efficiency and proposes actionable insights for improving practices in the sector.

**Abstract:**

The production efficiency factor is widely used to measure the zootechnical performance of a batch of broilers. The unit cost of production brings new elements to improve efficiency evaluation and financial sustainability for this activity in agriculture. This research aims to evaluate the production efficiency level of the crop to maximize the return on investment. This study uses Data Envelopment Analysis (DEA) with the computational processing of the SIAD software (Integrated Decision Support System). The variables selected were poultry housing, age at slaughter, feed consumed, mortality, and unit cost. The chosen output variable was the total available weight. The analysis spans 31 decision-making units (DMUs) composed of integrated producers, unveiling a frontier of efficiency delineated by the most exemplary DMUs. Notably, only two DMUs, specifically DMU 4 and DMU 23, approached the threshold of maximum relative efficiency. This research illuminates the critical role of unit cost in enhancing the assessment of production efficiency and financial sustainability within the agriculture environment. By setting benchmarks for efficient management and operational protocols, our findings serve as a cornerstone for improving practices among less efficient DMUs, contributing significantly to the literature on agricultural efficiency and offering actionable insights for the poultry farming sector.

## 1. Introduction

The poultry agro-industry in Brazil relies heavily on integration contracts as its organizational backbone [1]. In this integrated production system, contract producers are tasked with poultry stock rearing, while the extensive processing agro-industry supplies them with feed, chicks, and veterinary services [2]. Integrated producers shoulder responsibilities for aviaries, utilities, labor, and management structures [3].

The financial investments in this industry are substantial, with a single modern shed capable of accommodating approximately 80,000 birds costing around BRL 3 million, excluding property acquisition expenses [4]. To finance such facilities, most producers turn to bank debt for support.

While these integration contracts offer benefits, they also significantly reshape the financial risk landscape for integrated producers. They effectively transfer the risks associated with price fluctuations in feed and live chickens from producers to the agro-industry. Pro-producer payments remain independent of market feed, chick, and chicken price variations [4]. Additionally, producers bear production risks tied to diseases or climate conditions that may impact performance, as their compensation depends on delivered production capacity rather than housing capacity. These known risks should be on the radar of integrated producers [5].

However, integration contracts introduce new risks that producers need to navigate. For instance, long-term capital investments by producers can tether them to specific agro-industry partners. They face counterpart risks as they invest in aviaries and commit to these partnerships. The agro-industry may encounter financial or management challenges that disrupt the expected accommodation cycles per year, leading to potential contract extensions and additional capital requirements from producers [6]. 

To help integrated producers make informed investment decisions, the agro-industry aims to provide more comprehensive information on the financial risks associated with integration contracts in the poultry farming sector [7].

The compensation of integrated producers is typically rooted in a production efficiency scoring system. When birds are delivered to the agro-industry, crucial data, such as the total live weight of the batch and the number of birds delivered, are meticulously recorded. The agro-industry calculates feed consumption, the number of chicks supplied, and the days required for chicken production [8]. A comprehensive performance metric, the “production efficiency factor” or PEF, integrates the agro-industry indices. PEF fluctuates with the squad’s mortality rate, with higher mortality reducing the overall survival rate. Feed intake also affects it, as healthy, stress-free chicks tend to convert more feed into meat than stressed chicks. Another influencing factor is daily weight gain (DWG) [9]. The DWG measures the relationship between the average weight of the chicken and the days necessary for its production [10].

Integration contracts commonly specify a monetary value per bird delivered, along with premiums or deductions based on mutually agreed parameters between the agro-industry and the producer. Generally, the higher the production efficiency factor, the greater the financial performance, resulting in enhanced remuneration for integrated producers [11].

Within the academic literature, the PEF, or its equivalents such as the Productive Efficiency Index (PEI) or European Productive Efficiency Index (EPEI), are widely employed to gauge the zootechnical performance of broiler batches and determine producer compensation. These metrics are derived through a weighted analysis of zootechnical performance indicators, encompassing daily weight gain, viability, and feed conversion [12]. If, on the one hand, for the integrated producer, the higher the factor of production efficiency, the better the remuneration, for the agro-industry, the lower the unit cost of production, the higher the return on investment in the working capital employed, since the chicks and feed correspond to about 70% of the total cost of production [13,14].

The crux of this work’s significance lies in its novel approach, including unit cost data for evaluating efficiency and financial sustainability within the poultry agro-industry. This study employs the Data Envelopment Analysis method to scrutinize production efficiency levels, supported by computational processing through the SIAD software.

While the concept of using DEA to assess the efficiency of poultry farms may not be entirely novel, the main contribution of our study lies in its specific application and the novel insights it offers. The conventional method of evaluating zootechnical performance through the PEF primarily considers factors like daily weight gain, viability, and feed conversion. However, our research extends the analysis by incorporating the unit cost of production as a critical factor in determining efficiency. This addition offers a more comprehensive evaluation of financial sustainability within the agro-industry, which, to our knowledge, has yet to be extensively explored in the existing literature. By integrating this financial aspect, our study aims to help poultry farmers maximize their return on investment, making it directly relevant and beneficial to the industry.

Additionally, our research identifies specific DMUs that exemplify efficient practices, setting a benchmark for disseminating efficient management protocols. This practical application of DEA results in actionable recommendations for improving the processes of inefficient DMUs, contributing significantly to the poultry farming sector’s advancement. Therefore, our paper contributes to the field by enhancing the traditional DEA approach and extending its applicability to address contemporary challenges in poultry farming efficiency and financial sustainability.

The overarching aim of this study is to assess the production efficiency within the agricultural sector, focusing on maximizing return on investment. This encompasses a series of targeted objectives: firstly, to evaluate the efficiency of integrated producers; secondly, to pinpoint the sources and extent of inefficiencies among these producers; and thirdly, to devise strategic production approaches that enhance efficiency and address identified inefficiencies through the adoption of best practices. This comprehensive approach seeks to offer a granular understanding of efficiency dynamics and to foster the implementation of effective strategies for sustainable agricultural production.

We structured this paper into six sections. Following this introduction, the second section delves into the materials and methods, exploring the problematic situation and axiomatic modeling. Section 3 is the theoretical background, offering a comprehensive literature review, and Section 4 elucidates the solution to the problem by incorporating unit cost data into the analysis. Section 5 presents a discussion, and Section 6 offers concluding remarks on the research.

## 2. Materials and Methods

Operational efficiency is one of the drivers of the strategic objective established in the company’s annual planning. In this sense, the Executive Board requested a team of internal experts for a project to maximize the production efficiency of the company’s integrated producers.

**Figure 1:**
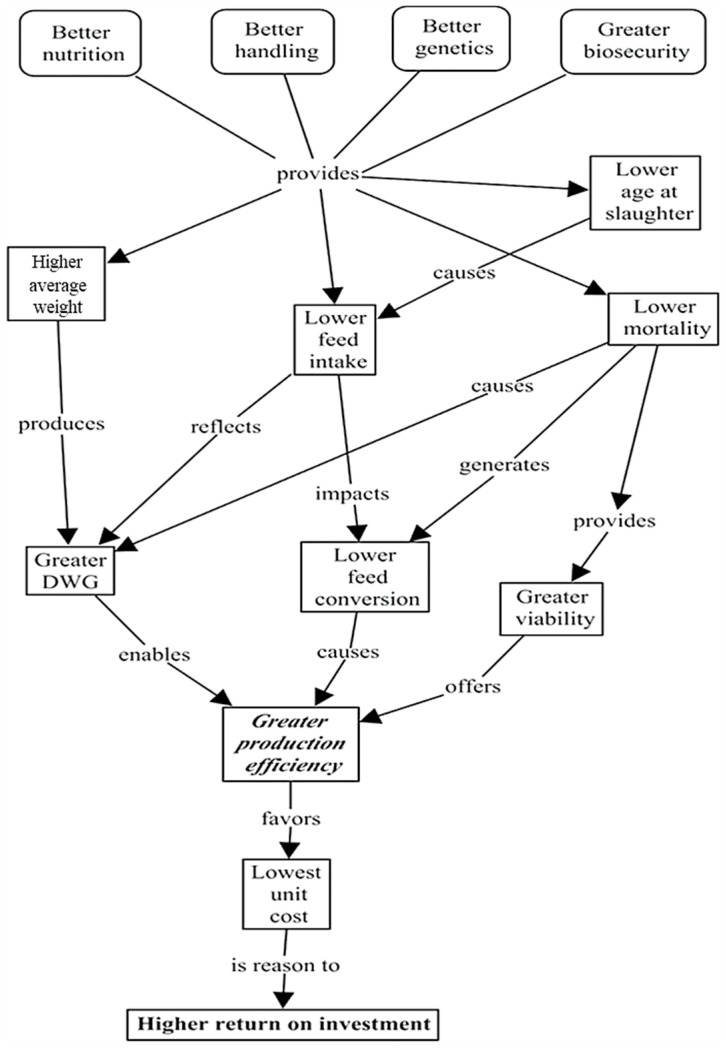
Cognitive map depicting the “maximize production efficiency” problem-solving approach.

We used the approach presented in [15] to construct cognitive maps. It has been defined as the “maximize production efficiency” problem label. We used the CmapsTools tool to consolidate the aggregate conceptual map with the individual perspectives of the company’s experts to show the step-by-step maximization of operational efficiency in the crop. We established a cause-and-effect relationship with hierarchies of the Primary Assessment Element (PAE) concepts. Finally, we used the auto-layout functionality to generate the format of the automatic cognitive map through the system’s algorithms. Figure 1 demonstrates the mental map of the problem.

As shown in Figure 1, the model seeks to achieve a higher return on investment in poultry farming through a cascade of interconnected factors leading to greater production efficiency. Better nutrition, handling, genetics, and biosecurity are foundational inputs that provide benefits such as higher average weight and lower slaughter age, directly contributing to improved production metrics. These improved practices lead to a lower feed intake and, consequently, a lower feed conversion ratio, reflecting a more efficient use of resources. Additionally, they result in lower mortality rates, thereby enhancing flock viability. The culmination of these factors translates into greater production efficiency. This efficiency, in turn, drives down the unit cost of production, ultimately favoring a higher return on investment, which is the primary goal of the production process.

The experts’ perception understood the problem as “How can agro-industry and integrated producers optimize the level of production efficiency to maximize return on investment?”. The general objective of this research was to evaluate the level of production efficiency of the crop to maximize the return on investment.

The secondary objectives were defined as follows:

1. Determine the production efficiency of integrated producers;
2. Identify the origins and level of inefficiency of integrated producers;
3. Establish production strategies that maximize efficiency and correct existing inefficiencies through good practices to be pursued.
4. Following the established objectives, the methodological structure will follow a case study process, identifying the need to evaluate and apply the technical model to an actual problematic situation. In addition to the research from a case study perspective, an exploratory literature analysis will also be considered, thus exposing the primary studies related to the theme.

### CCR Model

In the CCR model, it is possible to calculate efficiency according to the equations presented in this section. Considering H productive units generating m products using n inputs, the efficiency of these units will be calculated as follows:

(1) $$Max\;Ef0=\frac{\sum_{i=1}^{m}\mu_{i}y_{ik}}{\sum_{j=1}^{n}v_{j}x_{jk}}$$

As efficiency is the measurement subject, the outcome must lie between 0 and 100% or 0 and 1. Therefore, it is appropriate to introduce a constraint into the model [16], subject to

(2) $$\frac{\sum_{i=1}^{m}\mu_{i}y_{ik}}{\sum_{j=1}^{n}v_{j}x_{jk}}\leq 1\;for\;k=1,2\ldots H$$

Another significant constraint is that the weights must always be greater than ε, so $\mu_{i}v_{j}\geq \varepsilon$, where ε is a non-archimedean number (infinitesimally close to zero). The programming model described above implicitly acknowledges that many solutions exist that satisfy the ratio with a result less than or equal to one due to its fractional nature. Consequently, Charnes, Cooper, and Rhodes [17] proposed a linear programming model by introducing an additional constraint, limiting the product of inputs by their respective weights to the value of one. Hence,

(3) $$Ef=\frac{\sum_{r=1}^{m}\mu_{i}y_{ik}}{\sum_{j=1}^{n}v_{j}x_{jk}}$$

It is possible to state that

(4) $$Ef=\sum_{i=1}^{m}\mu_{i}y_{ik}-\sum_{j=1}^{n}v_{j}x_{jk}\leq 0$$

By making the denominator (virtual input) of the fraction above consistently equal to one, one can obtain the weights that maximize efficiency within this range of values. In this way, the proposed programming model transforms into

(5) $$Max\;Ef_{k}=\sum_{r=1}^{m}\mu_{i}y_{ik}$$

Subject to

(6) $$\sum_{j=1}^{n}v_{j}x_{jk}=1\;\sum_{i=1}^{m}\mu_{i}y_{ik}-\sum_{j=1}^{n}v_{j}x_{jk}\leq 0\;for\;k=1,2,\ldots H\;\mu_{i},v_{j}\geq \varepsilon$$

This programming model is input-oriented and referred to as the DEA model in multiplier or primal form, which allows the assignment of a set of weights to products and inputs, with the weights as decision variables. The CCR model will always represent the efficiency frontier as a straight line from the origin.

The BCC model defines efficiency by the following linear programming model, where the elements used in the model construction have the same meaning as when used in the CCR model, except for the added μ in the first equation.

(7) $$Max\;Ef=\sum_{i=1}^{m}\mu_{i}y_{i0}+\mu$$

Subject to

(8) $$\sum_{j=1}^{m}v_{j}x_{j0}=1\;\sum_{i=1}^{m}\mu_{i}y_{ik}-\sum_{j=1}^{m}v_{j}x_{jk}\leq 1\;for\;k=1,2,\ldots H\;\mu_{i}v_{j}\geq \varepsilon \;Without\;sign\;restriction$$

This model is already linearized, following the same process applied to the CCR model.

## 3. Literature Review

DEA is a nonparametric method used to evaluate the efficiency of decision-making units in converting inputs into outputs on a linear piecewise frontier constructed by the DEA model [18]. Building a piecewise linear surface to envelop data points, DEA identifies a frontier against which the performance of each DMU is measured, allowing for the assessment of relative efficiency without the need for a predefined functional form of the production function [19].

This technique is particularly adept at handling multiple inputs and outputs, making it invaluable for complex environments where the efficiency of entities, such as businesses, hospitals, or farms, is to be analyzed. Moreover, DEA offers insights into potential areas of improvement for inefficient DMUs by projecting them onto the efficient frontier, providing a roadmap for resource optimization and strategic planning. Its application spans from economics to engineering, highlighting its versatility and the growing recognition of its importance in operational research and performance management, as expressed in this study for specific analysis in poultry farming environments [20].

The theoretical basis was developed from the perspective of three thematic axes: (1) aviculture, (2) efficiency and zootechnical performance, and (3) Data Envelopment Analysis—DEA. The academic literature on zootechnical performance is extensive and quite comprehensive. We found that much research aims to maximize production efficiency.

**Figure 2:**
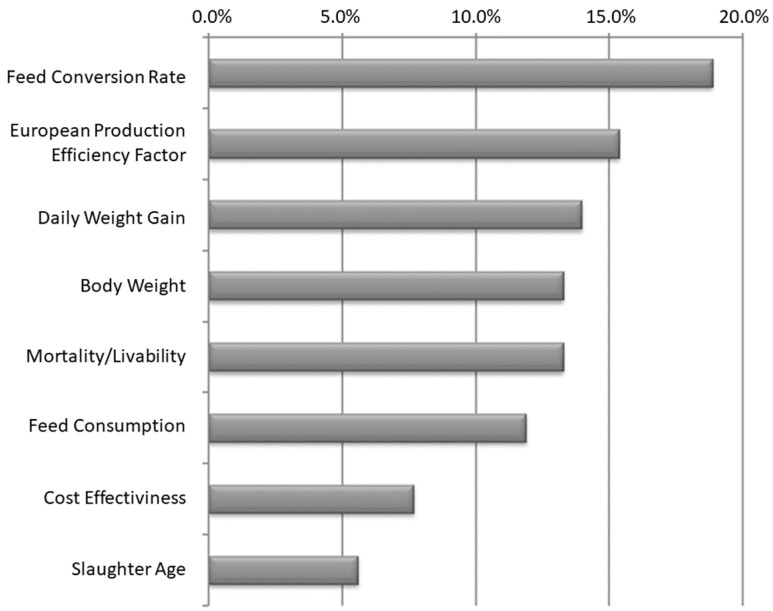
Frequency of primary metrics in aviculture research—data consolidation.

We researched theses, dissertations, national academic papers, and international academic works in Scopus based on zootechnical performance in aviculture. We reached the leading indicators used in the research based on the selected sample. Figure 2 consolidates the tabulation of the frequencies of the primary metrics used. We used a good part of these indicators as variables in the proposed model, and they are aligned with the indicators mentioned by the company’s internal experts in structuring the problem.

Yusuf [21] examined the technical efficiency of poultry egg production using Data Envelopment Analysis (DEA). The results show that the production of poultry eggs is profitable in the studied area and that most farmers are relatively efficient in the use of resources. Large-scale farmers are the most technically efficient, followed by medium-scale farmers, while small-scale farmers are the least efficient. The experience and education of farmers have a positive effect on technical efficiency, while family size has a negative impact [22].

Intending to analyze ways to improve the efficiency of chicken meat production in Bangladesh to meet the per capita demand, Begum [23] conducted a data analysis of 100 commercial poultry farms in 2007. Using Data Envelopment Analysis, it was possible to identify technical, allocative, and economic inefficiencies in poultry production in Bangladesh. Under the constant return-to-scale (CRS) model, the technical, allocative, and financial efficiencies were 88%, 70%, and 72%, respectively, while under the variable-scale return (VRS) model, these efficiencies were 89%, 73%, and 66%. The Tobit regression indicated that producer education and training are the most critical factors in increasing the efficiency of chicken production in Bangladesh. These findings are valuable to policymakers and extensionists to guide policies to increase production efficiency.

Heidari [24] analyzed the efficiency of farmers in identifying wasted energy uses and optimizing energy intake for broiler production. The author collected data from 44 chicken farms in six Yazd province (Iran) villages. Using Data Envelopment Analysis, the researchers determined the technical efficiency, pure technical efficiency, and scale efficiency of energy use in chicken farms. The DEA CCR and BCC models indicated that 10 and 16 farmers, respectively, were efficient. The mean technical efficiency values are pure technical efficiency, and they found the scale efficiency to be 0.90, 0.93, and 0.96, respectively. The results also showed that about 11% of the total input resources could be saved if farmers followed the input package recommended by the DEA.

Keramidou [20] conducted an efficiency analysis of the Greek poultry industry from 1994 to 2007 [25]. DEA was applied to obtain consistent inferences in nonparametric measurement performance. The results show that a loss of competitiveness in the Greek poultry industry accompanied a high overall inefficiency, which increased from 11% in 1994 to 14% in 2007. According to this study, the increase in inefficiencies in Greek poultry companies is mainly due to the persistent managerial inability to fully exploit the potential of technology and human resources. The analysis of a company’s efficiency by size categories shows that small businesses were not lower than larger ones in performance.

Begum [26] aimed to determine the efficiency of poultry farms in Bangladesh and to evaluate the influence of the agriculture system by contract using a Data Envelopment Analysis. Seventy-five commercial poultry farms were randomly selected. The Tobit model was used to regress the efficiency scores in some human capital and agricultural system variables to explain efficiency variations between the sample farms. The study also estimates elasticities to provide information on the magnitude of the influence of variables on technical efficiency (TE), allocating efficiency (AE), and economic efficiency (EE). The results show that the hiring system is positively and significantly related to the farm’s TE, AE, and EE. Empirical results can provide crucial information for policymakers seeking to improve the efficiency of poultry farming.

The study by Mahjoor [27] aimed to evaluate the technical, allocative, and economic efficiency of chicken farms in Iran using the Data Envelopment Analysis method. The authors assessed fifty-nine farms with increasing scale returns and 16 with decreasing returns. The results indicate that, on average, the farms’ technical, allocative, and economic efficiencies were 82%, 70%, and 57%, respectively, under the specification of CRS and 82%, 73%, and 64% under the specification of VRS. The analysis of factors associated with inefficiency revealed that education, producer age, training, and association with cooperatives of chicken producers are statistically significant factors related to technical, allocating, and economic inefficiency.

Rahimi [28] produced a study to evaluate the efficiency of a broiler farm using a new methodology. The methodology comprises DEA and some data mining techniques, such as artificial neural networks, decision trees, and cluster analysis. For the case study, the authors collected data from 22 poultry companies in Iran. They performed model validation using the k-fold cross-validation method. After evaluating the efficiency of each decision-making unit and its classification, they applied a model and presented decision rules to optimize the technique accurately and accurately.

The DEA method was used by Zhu and Qin [29] to evaluate the technical efficiency of large-scale laying in China, specifically the influence of mechanization on efficiency. The study found that large-scale laying still needs to be more mechanized, but mechanization has excellent potential for improvement, with mechanized creation being more efficient than traditional. Mechanization can effectively improve the technical efficiency of laying hen swellers and facilitate the development of standardized and large-scale laying improvement. The article also observed that the number of farmers with good educational training and reproduction training is directly proportional to the scale, region, and technical efficiency of large-scale laying hens and that the number of jobs in laying hens is in reciprocal proportion to the technical efficiency of laying. Therefore, the article recommends that governments promote the mechanization of applying hen farming and continue improving chicken farm owners’ and decision-makers’ quality and breeding skills.

In Ardabil Province, Iran, Amid [25] analyzed energy efficiency in broiler production [30]. The authors used the nonparametric method of DEA to separate efficient broiler producers from inefficient ones and calculate energy waste. The results indicated that the total energy use was high and fuel participation was the highest of all farm inputs. In addition, the results revealed the great potential to increase energy efficiency following the recommendations for energy efficiency.

Payandeh [31] assessed the environmental impacts of broiler production on 90 farms in Iran’s Isfahan province. They identified inefficient farms using DEA and proposed improvements to reduce energy and resource use and thus reduce environmental impacts. The authors calculated eleven ecological impacts using the life cycle evaluation method. The results showed that lowering farm energy use by 10.16% is possible. Food production was the main contributor to environmental impacts in almost all indicators. Improvements in energy use have reduced environmental impacts by up to 57%. The study showed that proper management of resources and energy could reduce environmental emissions in broiler production.

Martins and Asunción [32] researched the importance of amino acids in the formulation of diets for broilers, as well as an approach to the general aspects of the first limiting amino acids for birds, which are methionine and lysine. As a result, the authors point out that the evolution of genetic improvements in broiler strains facilitated significant gains in weight gain, breast yield, and feed conversion, making industrial aviculture one of the most competitive production chains in the country. Since nutrition is responsible for the largest share of production costs, formulating balanced diets is crucial to providing maximum food efficiency and better production performance.

Mitre [28] conducted a study on the effect of drinking water magnetization [33] to evaluate the critical factors of poultry production, such as the feed conversion rate, weight, feed intake, and viability. The results show that the magnetization of water for broilers fed a nutritional program that meets their daily needs was not beneficial.

Ilham [34] analyzed the supply chain efficiency of small laying hens in four regions of Indonesia. The author collected data from 139 respondents, including representatives of institutions, merchants, farmers, and managers of supermarkets and hotels. The author performed an efficiency analysis using DEA. The results showed that the involvement of farmers, the proportion between profit and cost of marketing, and the number of actors involved influence supply chain efficiency. Those with higher capital must shorten the supply chain by selling directly to consumers. The Indonesian Farmer Shop suggests increasing farmers’ incomes and stabilizing prices.

Intending to measure the relative efficiency of the intermediate poultry trader, Purwaningsih [35] used Data Envelopment Analysis and made recommendations based on the results of the DEA. The data processing result shows that three of eleven intermediaries could have been more efficient. These groups of intermediate traders should increase their relative efficiency by aligning input variables that influence the cost of distribution and overall cost.

Hatzizisis [36] determined the technical efficiency (TE) and scale efficiency (SE) of broiler farms in the Epirus region of Greece, calculating the margin at which farms can increase production based on specific amounts of inputs. The study was conducted on 110 farms randomly selected using Neyman’s stratified sampling method. The TE and SE of the decision units were determined using a production-oriented DEA approach. The results showed that the production system of chicken meat applied in Epirus is intensive, extensive, and characterized by high capital investments, high amounts of feed used, and a high efficiency rate. The authors highlighted the cost of feeding and the feed conversion rate as variables strongly related to TE and SE. Additional increases in efficiency should be focused on improving the feed conversion rate and the use of infrastructure and equipment in general.

To evaluate the effect of feeding and climate conditions in chicken coops on production performance in a commercial poultry company, Küçükönder [37] proposed a hybrid approach. This approach integrates DEA and Grey Relational Analysis (GRA). In this hybrid approach, the authors determined efficacy by the DEA method, and a performance classification was performed between effective months by the GRA method. It investigated whether the results of different Multi-Criteria Decision-Making (MCDM) techniques combined with the data fusion technique support the results of the proposed hybrid approach. The authors applied the study to the monthly production of a commercial company with 8000 Lohman Brown chickens. January, March, October, November, and December were the months with high production performance. When these months were ranked among themselves, January, March, and November were the top three. It is possible to use the proposed hybrid approach to determine the optimal production performance of the company, with temperatures between 20.25 °C and 26.41 °C, a moisture ratio between 47.60% and 54.25%, and the amount of feed per hen between 98 and 128 g.

A literature review [38] elaborated a meta-analysis based on a survey of articles published over 15 years on the factors affecting broiler production. The results found by the authors showed that the factors that most influenced the performance of broilers were temperature, wind speed, and genetic lineage.

Ibrahim [39] describes goal-setting models that accommodate desired output objectives or available inputs while improving efficiency in Data Envelopment Analysis. These models ensure efficient goals when inefficient or weakly efficient units want to expand or reduce outputs/inputs in cases of inbound/outbound redistribution or non-discretionary variables in a production system. This study presents two empirical studies that apply these models in the poultry production chain and the water, energy, land, and food sectors. The results obtained support the proposed models.

Van Limbergen [40] conducted a study investigating risk factors for health and performance in conventional broiler farms. As a result, the authors associated the following factors with increased mortality: tread quality, chick quality, type of ventilation, and rearing facilities. The factors related to performance were the sex of the birds, the health of the herd, control of the light intensity, the type of ventilation, daily food checks, the water system, and the type of feeding. They identified the initial mortality factors: daily gain, kind of adaptation to light, and type of water system. They found risk factors for the conviction rate: type of drinking system, daily gain, feed withdrawal time, type of ventilation, shed size, and type of feed.

In a study on the zootechnical performance of broiler chickens in different facilities, Borotto and Freitas [41] researched three other models of poultry facilities, enabling the better zootechnical performance of birds. The results obtained in the study demonstrated significance for the variable of daily weight gain, in which the yellow curtain presented a higher mean and feed conversion than the animals housed in blue and dark curtains. The variables of mortality and total convictions were not significant.

Caldas [42] developed research to evaluate the zootechnical performance of broilers according to the hosted genus and analyze the financial result of the activity under different governance mechanisms. The zootechnical lot indicators were better for males than those observed for mixed poultry rearing and female lots, respectively. However, when comparing the results with the expected values for the lineage, the genetic potential of the birds could have been better utilized by the production units. Regardless of the genus of the birds, economic analyses indicated that only broiler rearing in the integration model could confer economic profit to the activity. Finally, the authors found that poultry rearing does not receive financial support for any governance structure evaluated without secondary revenue from selling poultry beds.

An evaluation of the economic efficiency of a broiler production system was directed by Piran [3] using DEA and an internal benchmarking approach. The study analyzed longitudinal data from six years and applied the concept of flow accounting of the Theory of Restrictions to structure the DEA model. The authors used the Critical Incidents technique to explore the impact of interventions on system cost efficiency. The results show that the production system could reduce 32% of the total cost per production unit if it used the DEA-suited balance of inputs. The study highlights the importance of internal benchmarking to explore the evolution of cost efficiency over time and provides valuable information to guide continuous improvement.

Parlakay and Çimrin [43] conducted a study in Hatay Province, Turkey, to measure the technical efficiency of broiler farms in the region using DEA. The authors collected data from 19 broiler farms and measured using the entry-oriented approach. Technical efficiency was calculated as 0.95 with DEA-CRS and 0.97 with DEA-VRS. Farms could produce at the same output level by reducing the ins by 5% and 3%, respectively. They observed that the operators’ experience negatively affects efficiency. Inefficient farms use labor excessively, suggesting that better work organization can be a measure to improve efficiency. They concluded that broiler farms in Hatay province operate efficiently in terms of inputs and economies of scale.

Ilham [44] analyzed the performance of small chicken egg farms in three Indonesian cities. The authors collected data through interviews with 50 farmers and 12 poultry stores at the study sites and performed efficiency analysis using the DEA method. At the same time, they assessed financial viability by financial analysis. The results indicated that the efficiency level in Payakumbuh and Sidrap was better than in Blitar due to the excessive use of agricultural inputs. Technical services of poultry stores and local agricultural extension workers were recommended, in addition to reducing feeding costs and better allocating corn to optimize the relationship between feed and egg prices. The study concluded that small-scale chicken egg production is financially feasible.

Myeki [40] conducted a study on the efficiency of chicken producers in South Africa, covering 64 producers in three districts. Myeki [45] studied the economic conditions arising from COVID-19, climate change, and the Russia–Ukraine conflict. The two-step Data Envelopment Analysis with input orientation was used to evaluate the efficiency of the producers. The results show room for improvement in technical, allocative, and cost efficiency, with 10%, 20%, and 28% margins for reducing inputs without affecting production. Most producers were women, and districts showed significant differences in efficiency scores, with essential determinants such as mortality rate, heating costs, and health investments. The results provide insights to rethink the country’s poultry sector in the face of current shocks.

Phonpawi [46] proposed a study to analyze the technical efficiency of broiler production in northern Thailand and the factors affecting this efficiency. Phonpawi [41] used secondary data on the broiler industry in 17 provinces in northern Thailand from 2015 to 2019. The authors found that all 17 provinces in northern Thailand had different technical efficiency indices, with Lamphun province showing the highest score and Phayao the lowest. The overall average technical efficiency of the 17 provinces was considered high. The factors that influenced technical efficiency were the level of education, season, and age of farmers, all related to Sustainable Development Goal (SDG) policies, such as quality education, responsible consumption and production, and climate action.

The efficient management of the use of agricultural inputs for poultry production and its costs is of fundamental importance for poultry because efficiency involves the need to obtain more products with the exact amounts of inputs or to use them in optimal quantities at the lowest possible cost considering the production technology used in the properties. In other words, production costs should be decreased, and productivity increased, to improve efficiency and ensure the survival of integrated producers and the agro-industry.

Forecasting techniques and Data Augmentation methods are instrumental in advancing the efficiency assessment in poultry farming using DEA. Accurate forecasting is fundamental in setting the stage for DEA models. It enables the precise identification of inputs and outputs that are crucial for assessing the performance and efficiency of poultry farming systems. It involves using historical data to forecast future performance, which is critical for selecting appropriate DMUs and allocating resources. Using sophisticated forecasting techniques, such as those outlined by the study in [47], enhances the quality of input data for DEA models, thus increasing the accuracy and reliability of the analysis. In this context, Data Augmentation methods come into play by enriching the dataset with additional information, simulating a more robust environment to test our DEA model, and improving the reliability of the results [48]. This methodological approach ensures a strong foundation for our efficiency assessments, offering actionable insights with greater confidence in their validity.

Papers like [31] underscore the importance of comprehensive data inclusion, especially in environmentally sensitive domains. The combination of advanced forecasting and Data Augmentation ensures that the poultry farming efficiency assessment is built on a robust foundation, providing a comprehensive view of the system’s performance. In addition, Mirmozaffari and Kamal [49] underline the versatility of DEA in various fields, highlighting the relevance of our research in poultry farming efficiency assessment. Likewise, ref. [26] illustrates the application of DEA in poultry farming, offering further support for the efficacy of DEA models in our research on efficiency assessment in the agro-industry.

Within the realm of DEA, two core models, CCR and BCC, are pivotal in our research. The CCR model, as discussed in [50], assumes that changes in inputs and outputs have a proportional impact on efficiency, making it valuable for assessing uniform scaling effects in poultry farming. On the other hand, the BCC model, discussed in [51], offers greater flexibility by considering variable returns to scale. This adaptability accommodates the diversity and complexity inherent in poultry farming, where production processes may not exhibit uniform scale effects. The effectiveness of these DEA models lies in their ability to evaluate the relative efficiency of DMUs while considering multiple inputs and outputs simultaneously. Papers such as [52] emphasize the applicability of DEA models in assessing various aspects of agro-industry, which aligns with the poultry farming efficiency assessment in our research. These DEA models empower us to gauge poultry farming systems’ performance comprehensively, considering various inputs and outputs, thereby delivering a valuable tool for efficiency evaluation in the agro-industry. The inclusion of [26] showcases the wide-ranging applicability of DEA models and further reinforces their effectiveness in our research context.

Diverse researchers have calculated efficiencies through Stochastic Frontier Analysis (SFA). SFA methodologies have also found application in assessing the production efficiency of poultry farms, as illustrated in a study by Ahmed Khan et al. [53]. Therefore, the selection between the stochastic frontier approach and the DEA approach for efficiency measurement hinges primarily on the research’s aims, the nature of the firms involved, and the data at hand [20]. While each technique possesses its inherent merits and drawbacks, the optimal choice is contingent on the research objectives, the specific characteristics of the firms, and data availability [39]. Given our modest sample size, we opted for DEA in our analysis.

Table 1 presents the key features and characteristics of various approaches used to evaluate poultry farming efficiency. These approaches draw upon concepts from aviculture, efficient zootechnical performance, and DEA. The table provides insights into the input and output variables considered, the DEA models employed, and the incorporation of exogenous variables in the second-stage regression. This comprehensive overview of diverse methods sets the stage for our research, guided by the CCR and BCC models. It is crucial to consider these methods and models within the context of the poultry industry, where efficiency is paramount for sustainable and profitable production. We highlight that not all studies conducted second-stage regressions, and for some studies, it is unclear whether the authors used exogenous variables in a second-stage regression. Table 1 shows the key variables and DEA models used in each study.

Throughout the literature review, we identified many factors that play crucial roles in determining the efficiency of poultry production. These studies predominantly leveraged the DEA methodology to assess various aspects of performance in poultry farming systems. It is important to note that while these investigations delved into an array of crucial variables, none specifically incorporated unit cost when evaluating production performance. This distinctive gap in the existing research landscape underscores our work’s novel perspective and contribution, emphasizing unit cost’s significance as a pivotal factor in poultry farming efficiency assessment.

It is possible to see in Table 1 that many authors previously applied classic DEA models such as CCR and BCC in similar studies. However, our research distinguishes itself by introducing a novel perspective on efficiency assessment within the poultry industry. Incorporating unit cost as an input variable is an innovative feature of our approach. This innovation is rooted in the recognition that unit cost holds significant relevance in financial sustainability and resource allocation, as it directly affects the profitability of poultry farms. The emphasis on unit cost aligns with a granular approach, allowing for more precise insights into cost management strategies.

Moreover, our choice of using unit cost as an input aligns with industry practices, where understanding the cost efficiency of each production unit is crucial for decision making. Focusing on unit cost, our study offers a comprehensive view of operational and financial efficiency, making it pertinent to poultry farming practitioners and agro-industry stakeholders keen on maximizing return on investment while minimizing costs. In this way, our research addresses a significant gap in the existing literature and presents a valuable contribution to poultry farming efficiency assessment.

## 4. Case Study

We evaluated the efficiency analysis of the DMU-producing chickens based on two integrated approaches. The first one included traditional zootechnical performance indicators (number of birds housed, age at slaughter, feed intake, mortality, and total available weight). The second approach added the total unit cost, a variable used in academic studies to evaluate production efficiency in poultry farming. A new vision of productivity by adding the total unit cost to traditional production efficiency parameters could provide an element that best represents the return on investment in working capital for agro-industry.

We used the criterion of fixing the purchase prices of chicks and feed costs for all batches of the integrated ones to enable the comparison of the expenses between the production units, since the almost daily oscillation of these inputs would make it impossible to make a fair comparison in a different time horizon between the units. All evaluated lots are mixed types, with males and females created together.

According to Khoshroo [54], Data Envelopment Analysis is a linear programming algorithm for the measurement of the efficiency of production units. Two models are considered classics in the literature. CCR, or constant returns to scale (CRS), uses continuous scale returns, meaning that variations in any inputs produce proportional output variations. BCC, or variable returns to scale (VRS), works with variable scale returns, thus replacing the premise of proportionality between inputs and outputs with the premise of convexity.

The input variables selected were (1) poultry housing, (2) age at slaughter, (3) feed consumed, (4) mortality, and (5) unit cost. In this regard, “The smaller, the better”. For the selected output variable, (6) the total available weight, “The bigger, the better”. The variables were selected based on the best judgment of the internal specialists and submitted to a new reassessment after reviewing the academic literature on the subject. We evaluated a total of 31 DMUs formed by integrated producers.

We present a brief conceptualization for a better understanding of the definition of variables. The lodging of birds is the number of birds destined for breeding. The slaughter age is the number of days of production of the birds. The poultry feed consumed is the number of pounds consumed in birds’ output. Mortality corresponds to the percentage of birds that die during the production cycle. The unit rate corresponds to all the costs necessary to produce 1 kilo of poultry.

The data were collected in the company’s expert systems and processed in the Tableau. The closed lots correspond to the period from January 2021 to October 2022. We used the four classical approaches to efficiency assessment through input-oriented Data Envelopment Analysis: (1) input-oriented constant returns to scale (CCR), (2) output-oriented CCR, (3) input-oriented BCC (variable scale return), and (4) output-oriented BCC.

**Figure 3:**
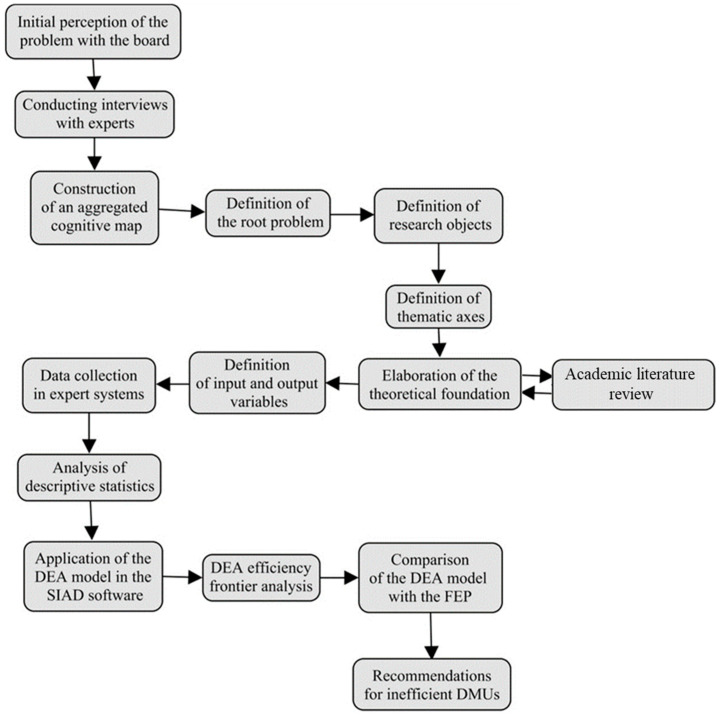
Step-by-step computational process using SIAD software for DEA and efficiency analysis.

We used the software SIAD (Integrated Decision Support System) for computational processing, available at http://tep.uff.br/softwares/ (accessed on 6 July 2023). The software uses DEA based on linear programming problems (LPPs) through the Simplex algorithm to find the efficiency of decision-making units (DMUs). With the algorithm, it is possible to obtain the CCR and BCC models oriented to input and output, with reverse borders, and has the possibility of placing restrictions on weights. The application provides complete results (efficiency indices, benchmarks, weights, clearances, and targets). The step-by-step study can be seen in Figure 3.

We must highlight that we also tested other input and output variables during the research cycle for consistency analysis and model validation. Variables were included and excluded. Some were tested as inputs and as outputs. In the model, we tested the zootechnical indicators of DWG (daily weight gain), FC (feed conversion), the PEF (production efficiency factor), and the quantity of birds produced. In the end, consensus with internal experts prevailed over the DEA model presented in this report.

### Results Analysis

The DEA model analysis reported the production unit efficiency and how much an unproductive unit (inefficiency < 1) needs to adjust its inputs and outputs to become efficient (efficiency = 1) compared to the sample DMU.

The characteristic of the DEA model presented is that it demonstrates the results obtained in evaluating efficiency based on the mathematical relationships of the variables used in the system and reports technical efficiency, which is a concept strictly related to the level of inputs and products. We have not included other important qualitative variables in the model. Table 2 exposes the data.

The notable variation coefficients for lodging, feed, and mortality, as reported in Table 2, indicate the diverse operational conditions and management practices across the evaluated decision-making units (DMUs). The high variation coefficient of 55.83% for lodging reflects substantial differences in the housing conditions provided for poultry, which may influence the birds’ growth and health outcomes. Similarly, the coefficient of 30.83% for feed indicates variability in feed consumption rates, possibly due to differing feed quality, feeding strategies, or the physiological characteristics of the birds. Mortality’s variation coefficient of 56.71% suggests discrepancies in health management and biosecurity measures among producers. These high coefficients of variation underscore the complexity of achieving uniform efficiency across integrated poultry farms and point to the potential for significant efficiency gains through the standardization of best practices in these areas.

The DMU is homogeneous with the materials and products. However, according to the internal experts aware of the reality of the producers, they are quite diversified in size, production technology, the technical knowledge of producers, property management practices, and level of commitment to the biosecurity of the process. The objective of this research was not to analyze the efficiency level of the possible production cluster. Table 3 exposes the correlation.

Table 3 presents the correlation analysis between the key variables considered in this study, offering insights into the interdependencies among factors influencing poultry farm efficiency. Notably, the table reveals significant correlations that are instrumental in understanding the dynamics of poultry production. For instance, the positive correlations between housing and feed indicate that better living conditions may be associated with higher feed consumption, possibly due to enhanced growth rates. Meanwhile, the negative correlations involving mortality suggest that factors leading to lower mortality rates may contribute to cost savings and improved weight outcomes. These correlations provide a quantitative foundation for this study’s objectives to identify inefficiencies and devise strategies for improvement. By understanding these relationships, we can better comprehend how changes in one aspect of production can influence others, thereby informing more targeted and effective interventions to optimize efficiency and productivity in integrated poultry operations.

From the data processed in the SIAD system, Table 4 demonstrates the values of inputs, outputs, and efficiencies for the CCR and BCC models, oriented to input and output for the 31 DMUs evaluated. The results of this research, demonstrating a frontier of efficiency of the best DMU with the best production practices, will be used as benchmarks for the dissemination of efficient protocols of management of property for the DMUs that present inefficiency, as exposed in Table 4. Only two DMUs (4 and 23) are at the border of maximum relative efficiency.

This research examined the CCR and BCC models, considering input- and output-oriented perspectives. We conducted this analysis to equip decision-makers with a more substantial and data-driven basis for making informed decisions.

The CCR model assumes constant returns to scale, meaning it does not allow for variations in scale efficiency. This simplicity can be advantageous when someone wants to assess overall efficiency without considering changes in the scale of operations. It provides a precise measure of technical efficiency, making it easier to interpret and implement.

On the other hand, the BCC model introduces variable returns to scale, allowing for flexibility in scale efficiency evaluation. This feature makes it more suitable for industries with significant scale differences among units. The BCC model accommodates variations in the production scale and can provide a more nuanced view of efficiency, mainly when some units may operate at different scales. The choice between CCR and BCC depends on the context and the level of variability in scale among the units under evaluation.

Considering that the input orientation for input variables in a DEA model offers the advantage of emphasizing the minimization of these variables to achieve efficiency. The model encourages the evaluated units to optimize resource usage, such as reducing feed consumption, mortality, and costs per production unit. Optimizing resource usage can be especially useful for identifying cost-saving opportunities and efficiency in poultry farming operations, aligning with current sustainability and waste reduction trends.

On the other hand, choosing an output orientation for the output variable, such as the total available weight, in a DEA model highlights the maximization of production. The output orientation is crucial for assessing farm production and performance efficiency. The emphasis on output maximization is vital for the poultry industry, where the primary goal is to achieve the highest possible bird weight. This emphasis helps to identify farms achieving a high production level relative to their inputs, assisting in decision making to optimize production and meet the increasing demand for broiler chickens. Therefore, choosing an output orientation for output is crucial for measuring efficiency in poultry farming.

A vital point observed is that the DMU with the highest efficiencies in any approach presented the lowest unit costs and mortality rates. We observed that in the CCR models oriented to input, the output of DMU 2 is considered efficient. In the BCC model introduced to input and output, we discovered nine efficient DMUs, demonstrating greater flexibility in results, characteristic of variable scale returns.

These DMUs are references for in-depth studies to identify success factors such as the management used, the types of facilities used, health protocols, management practices, and people management, for example, to disseminate these success cases. In the DEA model proposed for a more detailed analysis of the efficiency level, we worked on the CCR approach with input orientation. This approach keeps the output fixed and seeks to minimize inputs.

Another form of evaluation provided by the SIAD software is the reversed border. It is possible to assess how inefficient a DMU is. It indicates a ranking of the worst management practices observed. This ranking takes place automatically in the software by exchanging the outputs for inputs and vice versa. This functionality performs restrictions on weights, since many can come with zero values. Combining this information, SIAD provides composite and standard efficiency that enable better results for discrimination. Concerning the composed results, it combines several underlying factors or indicators into a single composite score. We present the results in Table 5.

We perceived with the results found in this research that the PEF, in addition to effectively showing the zootechnical performance indicators, also reflects, with reasonable precision, the financial results generated by the integrated producers, since the analysis of efficiencies of the DMU by the DEA model that had good performance also showed the lowest total unit costs and the best PEF. We perceived a relationship between high PEF, high efficiency, and low total unit costs. This perspective based on the data supports the company’s decision-making process and reflection points of change for better operational performance.

Table 6 demonstrates the strategies and opportunities for improvement so that the DMU achieves a more advanced efficiency level. The critical points are highlighted in red and should be worked on by the DMU. Viability is related to bird mortality. DWG is how much weight the bird gains per day. It is a relationship between the average weight of the bird and the number of days required for slaughter.

Moreover, feed conversion is a relationship between bird weight and feed intake. The bird is expected to convert feed into pounds of meat efficiently. Inefficient DMUs should seek to achieve the results of DMU 4.

We resume the points of attention identified in the interviews with specialists and validated by the theoretical foundation and highlight water quality, drinking systems, floor quality, ventilation, lighting, temperature, feed quality, pin quality, humidity control, adequate sanitary voids, control of entrances to farms, protection by green areas, the disposal of dead birds, evaluations of bird behavior, and interventions with drugs at the correct time to name a few examples of critical factors for maintaining high efficiency and productivity rates. We highlight that these statements are associated with this study’s company, providing different results when using other years of data or other companies with various performances.

Additionally, as technical recommendations, the Brazilian Agricultural Research Company (EMBRAPA) considers a good biosecurity program; among the main factors to consider, the following stand out: care in the acquisition of chicks; managing the location of the farm, access, and flow of traffic on the farm; feed and water care; sanitary management procedures; vaccination; hygiene; and the destination of discarded carcasses.

The agro-industry is expanding the use of management practices related to improving the biosafety and health of birds. However, poultry farming is financially risky. Even large and relatively efficient integrated producers have a relatively low average return on investment and time-consuming payback compared to the economy as a whole. Adequate levels of efficiency are primary conditions for survival and longevity in the food business. The profit margins are reduced, fixed and rotating investments are high, and the production volume needs to be scaled to seek lower costs.

One limitation of the model is that the efficiency presented in the DEA model is relative, which means that a DMU is efficient compared to the other DMUs in the sample. This same efficient DMU in the proposed DEA model may need to be more efficient than a DMU from another, more efficient, sample. The DEA model only works for this analyzed sample.

Finally, it is worth highlighting the limitation of the DEA model in providing a technical efficiency score. Taking into account a broader concept of efficiency, however, it can be concluded that an efficient poultry production system meets its objectives by using its resources in the best possible way to produce with low cost, animal welfare, biosafety, and sustainability in broiler production, which we did not address in this research.

## 5. Discussion

DEA is a robust tool to evaluate efficiency in agricultural production, particularly in poultry farming, where maximizing productivity and minimizing costs are crucial. This study highlights the importance of DEA in identifying exemplary practices and setting benchmarks for the industry. Compared with previous studies, the originality in including the unit cost as a crucial variable stands out, providing a more comprehensive perspective on economic efficiency beyond purely productive efficiency. Coelli [55] pointed to DEA as a valuable methodology to identify productive inefficiencies, but integrating financial aspects raises the analysis to a new level.

The agreement of the results obtained with previous research evidences the consistency of DEA as an analytical methodology, but its application in this specific context, which is focused on unit cost, introduces a new dimension for evaluating efficiency. This reinforces the relevance of cost management and production efficiency as pillars for sustainability in the poultry sector. In addition, the approach used in this study can serve as a model for future investigations in other areas of agriculture, as suggested in [56], which highlights the flexibility and adaptability of DEA in various productive contexts.

The application of DEA illuminates the performance spectrum of DMUs, with only two reaching near-optimal efficiency levels. This achievement corresponds with the first objective, providing a precise measure of production efficiency among integrated producers. Addressing the second objective, this study successfully identified the origins and levels of inefficiency. It revealed a substantial variation in efficiency across the DMUs, pinpointing specific areas where improvements are needed. The benchmarking process highlights the best practices at the most efficient DMUs, which can now serve as a model for others. For the final objective, this study established strategic production avenues that could bolster efficiency and rectify existing inefficiencies. The analysis suggests that others can significantly improve their production processes by emulating the operational protocols and management strategies of the most efficient DMUs. This strategic insight is crucial, as it identifies inefficiencies and provides a concrete pathway for improvement, aligning with the goal of maximizing return on investment through enhanced production efficiency.

The practical implications of these findings for poultry producers are significant, offering a roadmap for resource optimization and the adoption of market-leading practices. By applying the strategies identified as the most efficient, producers can improve not only their competitive position but also the environmental sustainability of their operations, aligning with the growing demands for sustainable production. These directions align with the recommendations in [57], which emphasize that the practical application of DEA results in the continuous improvement of production processes.

Although this study has limitations, it paves the way for future research. Including additional variables, such as environmental impacts and animal welfare, could enrich the analysis, offering an even more holistic view of efficiency in poultry production. Such methodological extensions could reveal additional insights into balancing production efficiency with environmental and social responsibility, a growing challenge for the sector.

In summary, this study contributes significantly to the literature on efficiency in poultry production by combining DEA methods with unit cost analysis. This innovative approach provides valuable insights for poultry production management and sets a precedent for future research. Adopting such efficient practices has the potential to transform the sector, promoting more sustainable and economically viable production in line with the expectations of a society that is increasingly aware of environmental and social challenges.

## 6. Conclusions

This research started with the question “How can agro-industry and integrated producers optimize the level of production efficiency to maximize return on investment?”. For this, the objective was to evaluate the level of production efficiency of the crop to maximize the return on investment. We achieved this objective by applying DEA based on linear programming (PPL) problems through the Simplex algorithm.

The results of this research, demonstrating a frontier of efficiency of the best DMU with the best production practices, will be benchmarks for the dissemination of efficient management protocols and property management for DMUs that present inefficiency. These benchmarks are a form of recognition for the excellent work performed. Only two DMUs (4 and 23) are at the border of maximum relative efficiency.

Finally, the limitation of the DEA model provided a technical efficiency score. Taking into account a broader concept of efficiency, however, it can be concluded that an efficient poultry production system meets its objectives by using its resources in the best possible way to produce with low cost, animal welfare, biosafety, and sustainability in chicken production, which we did not address in this research. Another area for improvement in our study is the absence of environmental impact variables. Integrating factors like carbon footprint and waste management could provide a more holistic perspective on poultry farming efficiency, aligning with sustainability goals. Future research should consider including these variables for a comprehensive assessment of economic and environmental dimensions in agriculture. Also, if variables such as the feed conversion rate, body weight, and daily weight gain return to statistical relevance in the model, they may be re-incorporated in this research, perhaps providing a more robust model. However, this condition only becomes valid through statistical performance.

This research’s key findings offer valuable insights for agro-industry managers and integrated poultry producers seeking to optimize production efficiency and maximize their return on investment. Using DEA models through the SIAD software has identified the most efficient DMUs and provides a benchmark for disseminating efficient management protocols. These high-performing DMUs, representing best practices, serve as exemplars for others in the industry. As only two DMUs (4 and 23) reached the border of maximum relative efficiency, there is evident significant room for improvement in most operations.

Furthermore, the concept of the reversed boundary, as implemented by the SIAD software, enables a comprehensive evaluation of inefficiency. It automatically identifies DMUs with suboptimal management practices, facilitating targeted improvements. This functionality, coupled with composite efficiency analysis, allows for a nuanced assessment of results, as by combining this information, SIAD provided composite efficiency, also called standard efficiency. We indicated a ranking with the worst management practices observed. We made the changed boundary evaluation in the software by exchanging outputs for inputs and vice versa. This functionality performed weight restrictions, since many can come with zero values. 

This research also highlights the broader concept of efficiency, encompassing low-cost production, animal welfare, biosafety, and sustainability. While we have yet to address these factors directly, it underscores the importance of optimizing resource utilization and aligning operations with these objectives. 

These findings suggest that agro-industry and integrated producers should focus on emulating the best-performing DMUs’ practices, addressing inefficiencies, and embracing a holistic approach to efficiency, including cost-effectiveness, animal welfare, biosafety, and sustainability. By using DEA and related tools, agro-industry managers can make better decisions to enhance their operations, improve financial sustainability, and contribute to the overall success of the poultry farming sector.

## Figures and Tables

**Table 1:** Summary of key features in studies assessing poultry farming efficiency using DEA.

Study	Input Variables	Output Variables	DEA Model Used	Exogenous Variables in 2nd-Stage Regression
Yusuf, S.A.; Malomo, O. [21]	Resources, Experience, Education, Family Size	Poultry Egg Production	DEA and OLS regression	Experience, Education, Family Size
Begum, I.A. et al. [26]	Resources	Poultry Production	CRS and VRS	Producer Education and Training
Heidari, M.D.; Omid, M.; Akram, A. [24]	Energy Use	Energy Efficiency	CCR and BCC	N/A
Keramidou, I.; Mimis, A.; Pappa, E. [20]	Resources, Technology, Human Resources	Poultry Production Efficiency	Bootstrapped DEA	Firm Size
Begum, I.A. et al. [23]	Resources, Agriculture System	Poultry Production	CRS and VRS	Farm Hiring System, Human Capital
Mahjoor, AA [27]	Resources, Education, Training, Cooperatives	Efficiency	CRS and VRS	Education, Producer Age, Training, Cooperation
Rahimi, I.; Behmanesh, R.; Yusuff, R.M. [28]	Various Metrics	Efficiency	DEA, data mining, artificial neural network, decision tree, and cluster analysis	N/A
Zhu, N.; Qin, F. [29]	Mechanisation, Education	Technical Efficiency	Tobit model	Education, Mechanisation
Amid, S. et al. [25]	Energy Use	Energy Efficiency	CCR and BCC	N/A
Payandeh, Z. et al. [31]	Environmental Impacts	Energy and Resource Use	CCR and BCC	N/A
Purwaningsih, R. et al. [35]	Efficiency Scores	Efficiency	CCR and CRS	N/A
Hatzizisis, L. et al. [36]	Feed consumption, labor hours per year, fattening days per breeding	Total produced quantity of live chickens per farm	CRS and VRS	N/A
Küçükönder, H. et al. [37]	Temperature, humidity, feed quantity	Chicken survival rate and egg yield	DEA-CRITIC-GRA methods	N/A
Ibrahim, M.D. et al. [39]	Growth, feed conversion, European production index	Dead on arrival and condemnation rate	Overall, the first week, after the first week	N/A
Myeki, L.W. et al. [40]	Feed, bird stock, bedding	Number of live broiler birds sold	CCR model	N/A
Pedolin, D.; Six, J.; Nemecek, T. [50]	Agricultural products	Environmental impacts	CRS model	N/A
Kouriati, A. et al. [51]	Land in acres, labor in hours, variable costs in EUR.	Each farm’s total amount of sales	CRS and VRS	N/A
Mirmozaffari, M.; Kamal, N. [49]	Number of institutions, beds, and health technicians	Numbers of outpatients and emergency visits, number of discharged patients	CRS and VRS combined with ADD, SBM ML, and MCDM	N/A
Mirmozaffari, M. et al. [47]	Energy consumption	Cement production	CCR, BCC, and FDH	Wastewater was removed, waste gas was removed, solid waste was removed, and pollution control was invested.

**Table 2:** Descriptive statistics of data.

Descriptive	Housing (m^3^)	Age	Feed (lb)	Mortality (%)	Cost (BRL)	Total Weight (kg)
Average	513,000	42	2341	5.10%	4.33	1504
Median	485,820	42	2270	4.56%	4.33	1446
Standard deviation	286,398	1	1.327	1.57%	0.10	869
Coefficient variation	55.83%	2.53%	56.71%	30.83%	2.23%	57.74%
Max	1,249,391	45	5940	9.15%	4.57	3956
Min	74,668	40	325	3.33%	4.06	195

**Table 3:** Correlation analysis between variables.

Correlation	Housing	Age	Feed	Mortality	Cost	Total Weight
Housing	1.00	0.13	1.00	−0.04	−0.36	1.00
Age		1.00	0.180	−0.04	0.31	0.17
Feed			1.00	−0.07	−0.36	1.00
Mortality				1.00	0.23	−0.07
Cost					1.00	−0.38
Total Weight						1.00

**Table 4:** Efficiency results.

DMU	Housing	Age	Feed	Mortality	Cost	Total Weight	CCR INPUT-Oriented	CCR OUTPUT-Oriented	BCC INPUT-Oriented	BCC OUTPUT-Oriented
1	544,436	43.13	2637	4.58%	4.36	1654	0.96	0.96	0.97	0.97
2	632,907	42.52	2913	3.82%	4.27	1899	0.97	0.97	0.97	0.97
3	359,794	43.92	1721	4.42%	4.42	1065	0.94	0.94	0.96	0.95
4	1,249,391	43.04	5940	3.49%	4.15	3956	1.00	1.00	1.00	1.00
5	359,318	40.90	1558	4.72%	4.33	1013	0.96	0.96	0.98	0.96
6	295,866	42.01	1352	4.73%	4.40	850	0.94	0.94	0.97	0.95
7	132,038	41.35	560	8.06%	4.50	347	0.91	0.91	1.00	1.00
8	254,029	43.02	1169	5.85%	4.33	736	0.94	0.94	0.97	0.96
9	359,864	42.95	1635	7.37%	4.23	1062	0.97	0.97	0.98	0.97
10	1,116,457	42.89	5143	7.27%	4.28	3334	0.97	0.97	0.99	0.97
11	646,762	41.26	2810	9.15%	4.39	1800	0.96	0.96	0.99	0.96
12	788,492	42.48	3487	8.92%	4.28	2276	0.98	0.98	0.98	0.98
13	303,212	42.22	1370	6.50%	4.24	903	0.98	0.98	0.99	0.99
14	762,851	41.06	3197	5.28%	4.33	2066	0.97	0.97	1.00	1.00
15	673,960	41.87	3108	4.25%	4.28	2017	0.97	0.97	0.98	0.97
16	717,801	42.02	3267	3.56%	4.33	2099	0.96	0.96	0.99	0.96
17	894,040	42.67	4213	4.56%	4.35	2656	0.94	0.94	0.98	0.95
18	606,406	43.67	2922	5.12%	4.34	1843	0.96	0.96	0.97	0.97
19	467,990	44.70	2263	3.70%	4.47	1370	0.92	0.92	0.95	0.94
20	326,485	43.31	1521	4.51%	4.42	944	0.93	0.93	0.95	0.94
21	779,931	43.02	3407	3.84%	4.30	2220	0.97	0.97	0.97	0.97
22	201,268	42.10	935	4.73%	4.30	603	0.96	0.96	1.00	1.00
23	227,720	39.85	956	3.56%	4.06	654	1.00	1.00	1.00	1.00
24	207,620	40.68	899	4.44%	4.33	579	0.95	0.95	0.99	0.96
25	336,127	41.63	1567	3.33%	4.28	1020	0.97	0.97	1.00	1.00
26	526,711	41.05	2275	5.00%	4.37	1446	0.95	0.95	0.99	0.95
27	758,505	42.70	3567	4.19%	4.30	2296	0.96	0.96	0.97	0.97
28	707,747	40.86	3071	4.30%	4.37	1951	0.95	0.95	1.00	1.00
29	485,820	43.07	2270	3.85%	4.28	1469	0.97	0.97	0.98	0.97
30	104,795	42.42	496	4.42%	4.42	308	0.93	0.93	1.00	1.00
31	74,668	43.53	325	6.65%	4.57	195	0.89	0.89	1.00	1.00

**Table 5:** Efficiency based on the CCR model, input-oriented.

DMU	Standard	Inverted	Composed	Composed *
1	0.959807	0.957755	0.501026	0.912693
2	0.97325	0.921659	0.525796	0.957815
3	0.935325	0.970419	0.482453	0.87886
4	1	0.902093	0.548953	1
5	0.962084	0.927591	0.517247	0.942242
6	0.935886	0.956157	0.489864	0.89236
7	0.907078	0.996113	0.455483	0.829729
8	0.939413	0.953986	0.492713	0.89755
9	0.966488	0.924736	0.520876	0.948853
10	0.972588	0.926735	0.522926	0.952588
11	0.956282	0.939747	0.508267	0.825884
12	0.976155	0.920622	0.527766	0.961405
13	0.979087	0.911807	0.53364	0.972105
14	0.966129	0.965768	0.500181	0.911154
15	0.969787	0.925749	0.522019	0.950935
16	0.960873	0.934861	0.513006	0.934516
17	0.944959	0.952938	0.496011	0.903557
18	0.959922	0.952485	0.503718	0.917598
19	0.92495	0.992131	0.466409	0.849634
20	0.92856	0.967473	0.480544	0.875382
21	0.974344	0.922017	0.526163	0.958485
22	0.963735	0.931735	0.516	0.939971
23	1	0.910863	0.544569	0.992013
24	0.948484	0.937486	0.505499	0.920841
25	0.973003	0.923468	0.524768	0.955942
26	0.946381	0.95268	0.496851	0.905087
27	0.964462	0.933268	0.515597	0.939237
28	0.949421	0.948954	0.500234	0.91125
29	0.968435	0.928197	0.520119	0.947474
30	0.930697	0.968508	0.481095	0.876385
31	0.885687	1	0.442843	0.806705

**Table 6:** Strategies and opportunities for efficiency improvements.

DMU	Viability	DWG	Food Conversion	PEF	Efficiency
4	96.51%	76.13	1.502	488	1.00
23	96.49%	74.59	1.463	490	0.99
13	93.50%	75.11	1.518	463	0.97
12	91.08%	74.01	1.532	440	0.96
21	96.16%	68.70	1.535	430	0.96
2	96.18%	73.25	1.534	459	0.96
25	96.67%	75.29	1.537	473	0.96
10	92.73%	74.68	1.543	449	0.95
15	95.75%	74.52	1.541	463	0.95
9	92.63%	73.78	1.539	444	0.95
29	96.15%	72.90	1.545	454	0.95
5	95.28%	72.20	1.538	447	0.94
22	95.27%	74.54	1.551	458	0.94
27	95.81%	73.86	1.553	456	0.94
16	96.44%	72.09	1.556	447	0.93
11	90.85%	73.63	1.561	428	0.93
24	95.56%	71.64	1.553	441	0.92
18	94.88%	73.15	1.585	438	0.92
1	95.42%	73.67	1.594	441	0.91
28	95.70%	70.36	1.574	428	0.91
14	94.72%	69.44	1.548	425	0.91
26	95.00%	70.23	1.574	424	0.91
17	95.31%	72.89	1.586	439	0.90
8	94.15%	71.33	1.588	423	0.90
6	95.27%	71.60	1.592	429	0.89
3	95.58%	70.41	1.615	417	0.88
30	95.58%	72.31	1.612	429	0.88
20	95.49%	69.79	1.610	414	0.88
19	96.30%	67.94	1.651	396	0.85
7	91.94%	68.62	1.616	390	0.83
31	93.35%	64.08	1.665	359	0.81

## Data Availability

Data are contained within the article.
